# Evaluation of Early Markers of Nephropathy in Patients with Type 2 Diabetes Mellitus

**DOI:** 10.1155/2016/7497614

**Published:** 2016-01-24

**Authors:** Pierina De Muro, Antonio Junior Lepedda, Gabriele Nieddu, Michela Idini, Hai Quy Tram Nguyen, Omar Lobina, Pietro Fresu, Marilena Formato

**Affiliations:** ^1^Department of Biomedical Sciences, University of Sassari, 07100 Sassari, Italy; ^2^Department of Diabetology and Metabolic Disease, AOU-Sassari, 07100 Sassari, Italy

## Abstract

*Aims*. T2DM often remains undiagnosed for many years because hyperglycemia develops gradually and may not produce any symptoms. As patients with T2DM are at increased risk of microvascular and macrovascular complications, the preclinical diagnosis of the state is the key point of the disease management.* Methods*. We evaluated parameters such as GAGs/PGs, NAG, and NGAL in urine samples from 43 normoalbuminuric T2DM patients and 31 apparently healthy control subjects.* Results*. The total urinary GAG excretion showed no significant differences between patients and controls. The electrophoretic analysis evidenced the presence of UTI and its degradation products (LSC and SM-LSC), CS, and HS. We observed modifications of HS and total UTI (including UTI and its degradation products) relative contents in T2DM patients compared with controls whereas no differences in CS percentage were found. NGAL levels were significantly increased in T2DM patients and were positively correlated with both NAG (*r* = 0.606, *p* < 0.0001) and the presence of hypertension (*r* = 0.352, *p* < 0.05).* Conclusions*. These data suggest that the assessed molecules could represent useful markers to detect early renal impairment in patients with T2DM.

## 1. Introduction

The chronic noncommunicable diseases represent one of the most difficult challenges for all health care systems, in both industrialized and developing countries, due to their continuous and relentless growth. The most paradigmatic example is certainly represented by diabetes mellitus. Epidemiological evidences suggest that, without effective prevention and control plans, diabetes will likely continue to increase globally [1]. In particular, T2DM often goes undiagnosed for many years because hyperglycemia develops gradually and may not produce any symptoms [2]. Since patients with diabetes are at increased risk of microvascular and macrovascular complications [3], the preclinical diagnosis of the state is the key point of the disease management.

The renal impairment in diabetes mellitus affects ~40% of type 1 and type 2 diabetic patients. Diabetic nephropathy is responsible not only for ESRD, but also for a significant increase in cardiovascular risk in this population. Although CKD is a common comorbidity condition of T2DM, in the early stages it is often unrecognized, especially in the elderly, and, therefore, untreated [4]. The high incidence of CKD highlights the importance of early diagnosis and treatment for delaying its progression [5]. Because of the increasing T2DM prevalence worldwide and the serious consequences of this disease on global health, it would be of great value to develop more useful and reliable (and less expensive) diagnostic tests. Theoretically, urine represents an excellent substrate for identifying both potential biomarkers for early diagnosis of nephropathy and more effective therapeutic targets or monitoring the progression of renal damage. UAE is currently the “gold standard” for detection or prediction of both diabetic nephropathy and cardiovascular risk, even though in T2DM its predictive power is probably limited to cardiovascular events rather than renal functional impairment. In addition, structural changes in the GBM may occur before the onset of microalbuminuria. In the early stages of diabetic nephropathy, characterized by a low urinary excretion of albumin, the charge-dependent glomerular permselectivity seems to be particularly affected [6]; these findings suggest an initial loss of functional groups in the GBM with a consequent increase in the pore size of the renal filtration barrier. This charge-dependent permeability of the GBM is probably due to the presence of anionic constituents, especially HSPG. It is therefore important to identify urinary markers that may offer greater sensitivity, earlier detection, and greater predictive power for diabetes complications to overcome limits of UAE tests.

Over the past few years we have described quali-quantitative variations of GAGs/PGs in both normoalbuminuric type 1 and 2 diabetic patients with respect to healthy control subjects, suggesting that both levels and relative abundance of urinary GAGs may be predictive of altered GAG metabolism in diabetes mellitus [7, 8]. Furthermore, we recently evidenced that UTI, a small PG found in urine, may represent a useful marker for monitoring kidney function in those patients at high risk of developing renal impairment [9].

It has been shown that tubular damage occurs early in the course of diabetic nephropathy and that it is not merely secondary to glomerular damage as previously thought [10]. From a clinical point of view, the excretion of tubular markers may have a higher predictive power compared to UAE. In particular, the concentration of NAG may even increase in the absence of elevated albumin excretion. In addition, NGAL is one of the earliest expressed molecules by renal tubular cells after insults of various origin. An increase of both serum and urinary NGAL has been described in diabetic patients before the onset of microalbuminuria, corroborating the recent theories of a phase of subclinical tubular involvement that anticipates the future appearance of the most well-known glomerular dysfunction [11].

Therefore, the aim of the present study is to assess parameters such as urinary GAGs/PGs, NAG, and NGAL to detect alterations of renal function in normoalbuminuric patients with T2DM.

## 2. Materials and Methods

### 2.1. Subjects

43 normoalbuminuric patients with type 2 diabetes mellitus, referred to the Unità Operativa di Diabetologia e Malattie del Ricambio, AOU-Sassari, were enrolled. Exclusion criteria were ketoacidosis, fever, infection, surgery as well as evidences of systemic diseases, renal, cardiac, or hepatic diseases, and malignant tumors. Normoalbuminuria was defined as a urinary albumin excretion rate lower than 30 mg/24 h (mean of three different samples over a period of three months). The mean age of the T2DM patients included (16 men and 27 women) was 64.21 ± 7.18 years and the mean value of known duration of diabetes was 7.02 ± 5.15 years. The patients had no complications at the time of the study, but 27 of them (62.8%, 8 men and 19 women) were hypertensive (systolic blood pressure ≥ 160 mmHg and/or diastolic blood pressure ≥ 90 mmHg) and were under antihypertensive treatment.  Table 1 shows the demographic and clinical characteristics of patients.

The control group was composed of 31 apparently healthy subjects matched for age, sex, and BMI with T2DM patients.

Informed consent was obtained before enrolment. Institutional Review Board approval was obtained. The study was conducted in accordance with the ethical principles of the current Declaration of Helsinki.

### 2.2. Urinary GAG/PG Analysis

Urinary GAG/PG purification and quali-quantitative analysis were performed by a method previously described [12]. Briefly, early morning urine samples (about 50 mL) were collected and, after centrifugation at 3,000 ×g for 15 minutes at 4°C, the sediment of broken cells or tissues and other solid materials was discarded. Clarified urine was applied to a column (Econo-Column Chromatography Columns, 0.5 × 20 cm, Bio-Rad Laboratories, CA, USA) packed with 6 mL of DEAE-Sephacel resin (GE Healthcare Life Sciences, UK), previously equilibrated with a buffer containing 0.02 M tris-HCl, 0.15 M NaCl (pH 8.6). After exhaustive washing, urinary GAGs/PGs were eluted with a buffer containing 0.02 M tris-HCl, 2 M LiCl (pH 8.6), and assayed for hexuronate content, using glucuronic acid as a standard. Hexuronate levels were normalized for urinary creatinine concentration, formerly determined by the Jaffè method (Sentinel CH, Milan, ITA). Urinary GAG/PG composition was determined by electrophoresis on cellulose acetate strips. The identification of GAGs/PGs was performed according to their comigration with standard GAGs and UTI purified from human urine (SCIPAC Ltd., UK) and to their electrophoretic profiles after enzymatic depolymerization with specific endoglycosidases. Images were acquired and analyzed by means of Gel Doc XR system and Quantity One software (Bio-Rad Laboratories, CA, USA). GAGs/PGs were expressed as relative percentages.

### 2.3. NAG and NGAL Determinations

Urinary NAG was estimated kinetically by using NAG colorimetric kit (FAR DIAGNOSTICS, Verona, ITA), whereas NGAL levels were performed with the Lipocalin-2/NGAL Human ELISA Kit (Abcam, Cambridge, UK). The assays were performed according to the protocols provided by the manufacturers.

### 2.4. Statistical Analysis

Statistical analyses were performed using the software package Sigma Stat 3 (Systat Software). Values are reported as mean (±SD) or median (interquartile range). Differences between groups were assessed by Student's *t*-test or the Mann-Whitney rank sum test when data failed the normality test. Association between the examined variables was investigated nonparametrically by Spearman's rank correlation analysis. The statistical significance was set at *p* < 0.05.

## 3. Results

**Figure 1:**
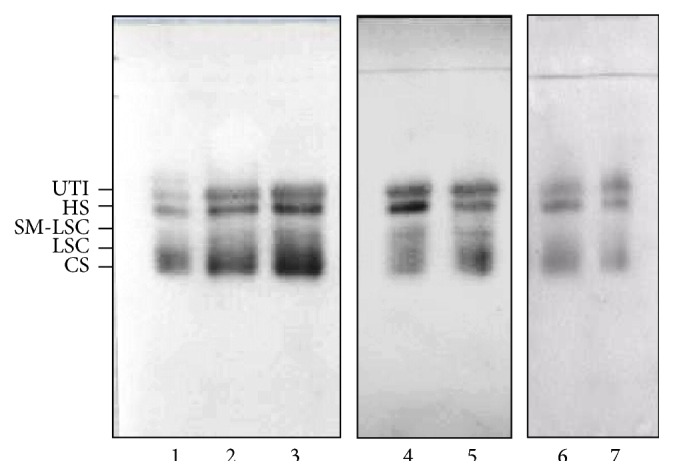
Representative cellulose acetate electrophoretic profiles of urine glycosaminoglycans/proteoglycans from T2DM patients (lanes 1–5) and control subjects (lanes 6-7). UTI: urinary trypsin inhibitor. LSC: low sulfate chondroitin sulfate. SM-LSC: slow migration-LSC. HS: heparan sulfate. CS: chondroitin sulfate.

**Figure 2:**
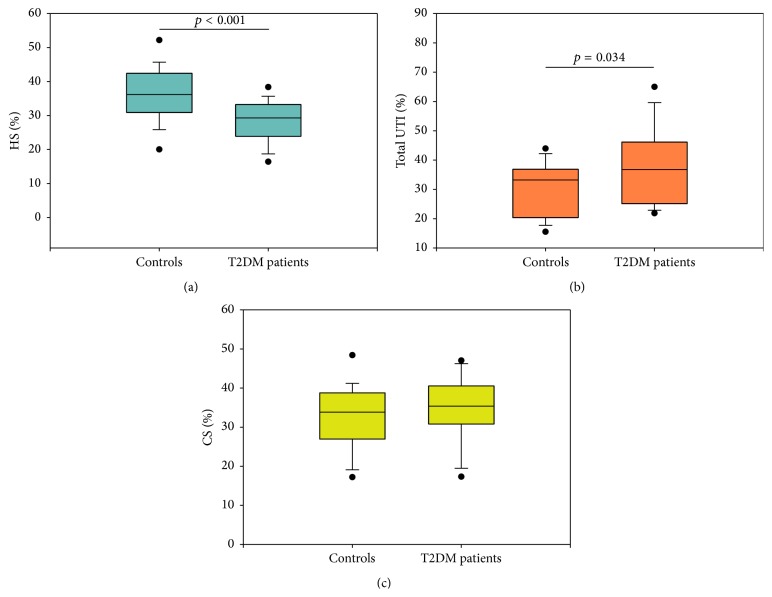
Plots showing the median (line within box), 25th and 75th percentiles (box), 5th and 95th percentiles (whiskers), and outliers (•) of HS (a), total UTI (UTI plus SM-LSC and LSC) (b), and CS (c) percentages in the controls group and in T2DM patients. Percentages were evaluated by performing image analysis on cellulose acetate electrophoretic profiles using Quantity One software (Bio-Rad Laboratories). Differences with *p* values <0.05 were considered statistically significant.

**Figure 3:**
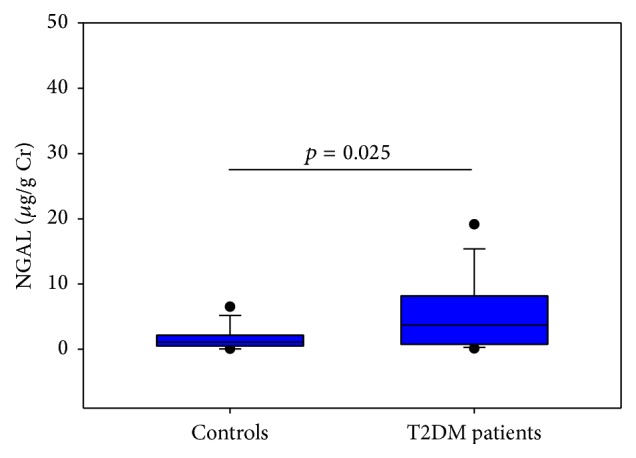
Plot showing the median (line within box), 25th and 75th percentiles (box), 5th and 95th percentiles (whiskers), and outliers (•) of neutrophil gelatinase-associated lipocalin (NGAL) levels, normalized for creatinine (Cr) content, in the controls group and in T2DM patients. Differences with *p* values <0.05 were considered statistically significant.

The total urinary GAG excretion showed no significant differences between patients and controls (3.757 (2.868–5.234) mg of hexuronate/g creatinine versus 4.40 (3.475–5.635) mg of hexuronate/g creatinine, *p* = 0.201). The electrophoretic profiles of urine GAG/PG samples (Figure 1) showed the presence of UTI and its degradation products (LSC and SM-LSC), CS, and HS. Following image analysis, a significant reduction of HS (*p* < 0.001, Figure 2(a)) and a corresponding increase of total UTI (including UTI and its degradation products) (*p* = 0.034, Figure 2(b)) were evidenced in patients, whereas no differences in CS percentage were found (*p* = 0.272, Figure 2(c)). The urinary excretion of NAG showed no differences between the two examined groups (3.547 (1.915–5.123) U/g creatinine versus 3.210 (2.000–3.835) U/g creatinine, *p* = 0.202), whereas the median value of NGAL was significantly increased in T2DM patients with *p* = 0.025 (Figure 3). The comparison among urinary levels of the evaluated markers and parameters such as age, BMI, duration of disease, and renal function indexes allowed us to detect significant relationships in T2DM patients among NGAL levels and both NAG (*r* = 0.606, *p* < 0.0001) and the presence of hypertension (*r* = 0.352, *p* < 0.05).

## 4. Discussion

The identification of sensitive markers of renal function continues to be of considerable interest. Although UAE is considered the “gold standard” for diagnosing renal dysfunction, other markers, detectable at an earlier stage of the disease, may have diagnostic and/or prognostic value. Moreover, the association between glomerular lesions and MA is less pronounced in T2DM. In this respect, Fioretto et al. [13] found structurally normal glomeruli in approximately 30% of microalbuminuric T2DM patients, whereas they did not evidence MA in patients with glomerulosclerosis. The known correlation between MA and cardiovascular events [14] can be explained by the fact that both microvascular and macrovascular complications in T2DM are also present in the kidney, making MA an index of generalized vascular pathology.

Several studies have been performed to elucidate the molecular mechanisms underlying glomerular ultrafiltration, suggesting a role of PGs, especially their carbohydrate moieties, in maintaining the glomerular filtration barrier [15]. In this respect, the glycosaminoglycan moiety of HSPG is thought to be involved in the pathogenesis of diabetic nephropathy [16]. The presence of PGs such as perlecan [17] and agrin [18] and collagen VIII [19] in the GBM is well documented. These molecules contribute to the negative charge of the GBM and may thus play a role on the permselectivity of the glomerular barrier. However, a study on mice lacking exon 3 of perlecan (HSPG2) that causes loss of attachment sites for HS side chains did not detect any kidney impairment, such as proteinuria [20]. This could be due to compensatory mechanisms, but, most likely, this observation further strengthens the hypothesis that the GBM alone cannot be responsible for the permselective properties of the glomerular barrier. PGs are present on the surface of endothelial cells and podocytes, as well as in the mesangial matrix cells. Cultured immortalized human podocytes express both mRNA and protein for syndecan-1, versican, and perlecan [21]. The negative charges on podocytes are thought to contribute to charge selectivity, podocyte stability, and signaling. In this cross-sectional study, we observed a significant reduction of HS relative abundance in T2DM patients with respect to healthy subjects. This reduction may be related to an alteration of the processes of HS synthesis and/or sulfation. In this respect, Van Det et al. [22] described a reduction of both synthesis and sulfation of HSPG by mesangial cells and glomerular visceral epithelial cells treated with high doses of glucose. Moreover, Yokoyama et al. [23] reported a reduced HS N-sulfation in the urine of T2DM patients.

The alteration of tubular reabsorption is found almost always in the early stages of kidney disease, including those characterized by predominant glomerular involvement. In this study, we observed an increase of NGAL in normoalbuminuric diabetic patients. Being an iron transporter, NGAL may be expressed by the damaged renal tubule to induce regeneration, since this element is necessary for reepithelialization. Furthermore, it is known that pathological changes in diabetic nephropathy involve accumulation of extracellular matrix, the degradation of which involves mainly MMPs. The activities of these enzymes are dependent on metal ions and are limited by TIMP-1. In particular, it has been reported that a high glucose concentration causes a reduction in the amount of MMPs secreted by the mesangial cells [24] and an upregulated expression of TIMP-1 [25], leading to extracellular matrix accumulation. NGAL may represent a universal activator of the MMP family [26]. In fact, it is known that NGAL modulates MMP-9 activity by protecting it from degradation [27], activating directly the MMP-9 precursor, and counteracting the inhibiting effect of TIMP-1 [26]. Thus, it may be suggested that NGAL is overexpressed to delay the progression of renal fibrosis in diabetic nephropathy, by preserving the enzymatic activity of MMP-9.

In our study, the urinary levels of NGAL show a highly significant relationship with the urinary NAG levels and a positive relationship with the presence of hypertension. We did not find any relationship among these parameters and serum creatinine levels or eGFR, probably because the increase of serum creatinine may not be evident before 50% or more of the nephrons are damaged.

Image analysis of GAG/PG profiles also enabled us to detect a significant increase in the relative content of total UTI in T2DM patients.

The role of inflammation in the pathogenesis of type 2 diabetes and associated complications is now well established. Multiple mechanisms underlie defective insulin secretion and responses in type 2 diabetes. These include glucotoxicity, lipotoxicity, oxidative stress, and endoplasmic reticulum stress. Interestingly, all of these mechanisms are associated with inflammatory responses [28].

During inflammation, UTI is released from I*α*I family proteins through proteolytic cleavage by neutrophil elastase in the peripheral circulation or at the inflammation site [29], therefore representing a positive acute phase protein. Furthermore, UTI is rapidly cleared from circulation by renal excretion into urine where it represents a useful inflammatory marker [7–9, 30].

In conclusion, we observed an increased level of NGAL, as well as an altered relative content of HS and total UTI in normoalbuminuric T2DM patients with significant correlations among NGAL levels and both NAG excretion and presence of hypertension. It is likely that, rather than a single biomarker, the identification of a panel of strategically selected analytes may represent a useful tool for the accurate prediction and monitoring of the disease. In this perspective, our results, although preliminary, suggest that the assessed markers could represent good candidates to detect early renal alteration in normoalbuminuric patients with T2DM. Further longitudinal studies with a larger sample size are required to clarify these results.

## Figures and Tables

**Table 1:** Demographic and clinical characteristics of both patients and controls.

Parameters	Controls	Patients
Age (years)	61.14 ± 6.65	64.21 ± 7.18
Sex (men/women)	10/21	16/27
BMI (kg/m^2^)	26.355 ± 2.247	27.583 ± 3.619
Duration of disease (years)		7.02 ± 5.15
Blood glucose (mg/dL)	88.26 ± 21.21	131.59 ± 33.40
HbA1c (%)		6.34 ± 0.73
Total cholesterol (mg/dL)	165.35 ± 24.38	170.71 ± 27.01
HDL cholesterol (mg/dL)	49.22 ± 10.21	55.20 ± 11.20
LDL cholesterol (mg/dL)	92.54 ± 13.58	96.24 ± 18.91
Triglycerides (mg/dL)	89.65 ± 36.28	94.47 ± 42.42
Serum creatinine (g/dL)	0.75 ± 0.12	0.78 ± 0.15
eGFR (mL/min/1.73 m^2^)		89.6 ± 14.5
Microalbuminuria (mg/24 h)		9.38 ± 6.67
Systolic blood pressure (mmHg)		132.1 ± 16.2
Diastolic blood pressure (mmHg)		78.6 ± 9.7

Data are mean ± SD. eGFR = estimated glomerular filtration rate calculated using the equation developed in the MDRD (Modification of Diet in Renal Disease) study.

## Abbreviations

T2DM: Type 2 diabetes mellitus
GAGs/PGs: Glycosaminoglycans/proteoglycans
NAG: N-Acetyl-*β*-D-glucosaminidase
NGAL: Neutrophil gelatinase-associated lipocalin
UTI: Urinary trypsin inhibitor
LSC: Low sulfate chondroitin sulfate
SM-LSC: Slow migration-LSC
HS: Heparan sulfate
CS: Chondroitin sulfate
ESRD: End-stage renal disease
CKD: Chronic kidney disease
UAE: Urinary albumin excretion
GBM: Glomerular basement membrane
BMI: Body mass index
MA: Microalbuminuria
HSPG: Heparan sulfate proteoglycan
MMPs: Matrix metalloproteinases
TIMP: Tissue inhibitor of metalloproteinase
eGFR: Estimated glomerular filtration rate.
